# Cadmium-induced structural reorganization and coordination evolution in SeTeSn chalcogenide glasses: correlation with photon interaction behaviour

**DOI:** 10.1039/d6ra02610a

**Published:** 2026-07-02

**Authors:** Vishnu Saraswat, S. D. Sharma, R. K. Chaudhary, Z. Khattari, Neeraj Mehta

**Affiliations:** a Department of Electronics and Communication Engineering, SR University Warangal Telangana 506371 India; b Medical Physics Section, Bhabha Atomic Research Centre India; c Department of Physics, Faculty of Science, The Hashemite University P. O. Box 330127 Zarqa 13133 Jordan; d Department of Physics, Banaras Hindu University Varanasi India dr_neeraj_mehta@yahoo.co.in

## Abstract

In this study, quaternary SeTeSnCd chalcogenide glasses (ChGs) for radiation shielding applications are investigated. The gamma-ray and X-ray attenuation properties of the prepared glasses were studied in a wide energy range by using a high-purity diode detector and standard radioactive sources. The linear attenuation coefficient (LAC) was evaluated for thicknesses in the range of 0.5–2.0 mm, and a comprehensive thickness dependence analysis was performed, which has not been widely reported for such systems. The experimentally obtained LAC values were used to estimate key shielding parameters, including transmission factor (TF), half-value layer (HVL), tenth-value layer (TVL), mean free path (MFP), and radiation protection efficiency (RPE). These parameters were also calculated theoretically using the Phy-X/PSD program to confirm. The results indicate that the radiation shielding performance is improved significantly by increasing the cadmium content in the SeTeSn glass matrix. The RPE approaches almost 100% at low photon energies, whereas it decreases at high energies. In contrast, TF, HVL, TVL, and MFP increase with photon energy. A comparison shows that the studied ChGs exhibit better shielding performance than conventional commercial glasses, such as RS 360, RS 253G18, and types A, B, and C. The shielding efficiency order is Cd6 > Cd4 > Cd2 > RS360 > RS253G18 > Type A > Type B > Type C. It is also interesting to note that the prepared glasses have higher RPE values than the commercial 10 mm-thick glasses, even at 2 mm. These findings suggest that the synthesized ChGs are promising candidates for radiation shielding in diagnostic imaging applications across the energy range of 20–150 keV, including mammography, dental radiography, conventional X-ray imaging, and computed tomography/positron emission tomography (CT/PET) systems.

## Abstract 2

In this study, quaternary SeTeSnCd chalcogenide glasses (ChGs) for radiation shielding applications are investigated.

## Graphical abstract

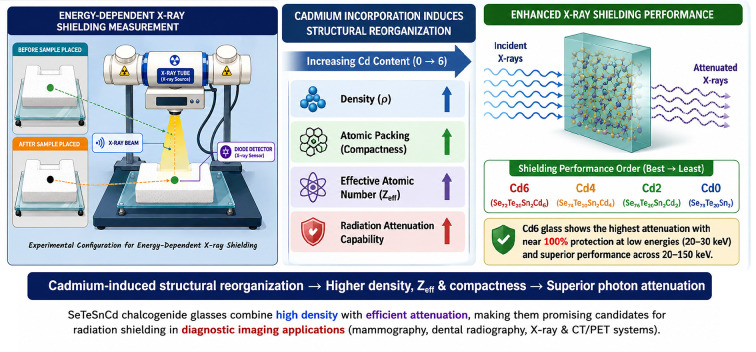

## 1. Introduction

Chalcogenide glasses (ChGs) based on selenium and tellurium have attracted considerable attention owing to their flexible network structures, high polarizability, and tunable electronic properties.^1,2^ The absence of long-range order in these materials allows significant structural rearrangement upon compositional modification, making them ideal systems for investigating coordination evolution in multicomponent amorphous networks. In Se-rich systems, the structure is generally described in terms of chain-like Se units and heteropolar Se–Te linkages, whereas the introduction of additional metallic species can modify connectivity by forming new bonding configurations. Prolonged or elevated exposure to such radiation can induce severe biological effects, including mutagenesis, carcinogenic outcomes, and cellular degradation. Beyond its health implications, ionizing radiation can disrupt geochemical balances and affect ecological systems by altering soil, water, and mineral compositions.^1–3^ To address these challenges, the development of advanced radiation shielding materials has become a research priority. The effectiveness of such materials is determined by multiple parameters, including their attenuation efficiency across a broad energy spectrum, structural stability under radiation exposure, non-toxicity, manufacturability, and economic viability.^2–5^ Importantly, materials intended for radiological protection must exhibit sustained integrity under prolonged irradiation without compromising environmental or biological safety.^4–7^ An effective shielding material must possess a high radiation absorption cross-section and demonstrate substantial attenuation of incoming rays over a minimal penetration depth (thickness).^7,8^ The choice of material depends mainly on the intended application. For instance, concrete, which effectively attenuates X-rays, is commonly used as an absorber in the exterior walls of X-ray rooms. However, while concrete may be suitable in certain scenarios, its tendency to crack and lose moisture under prolonged radiation exposure necessitates exploring alternative materials.^7–10^ Disordered materials are widely recognized for their strong radiation attenuation capability, which arises from their relatively high density and complex atomic-scale arrangement. The presence of a large number of atoms per unit volume increases the probability that incoming radiation will interact with the material, thereby reducing its transmission efficiency.^8,9^ This behaviour is particularly important for shielding against X-ray and gamma-ray photons. In glassy systems, the absence of long-range crystalline order leads to a random distribution of atoms. Such an amorphous structure creates numerous interaction sites, allowing radiation to undergo repeated scattering and absorption events as it passes through the material.^11–13^ Consequently, glasses provide an effective barrier against penetrating radiation.

In recent years, extensive efforts have focused on investigating various glass families, such as phosphate, silicate, borate, and tellurite glasses, to improve their radiation-shielding performance. These materials are commonly fabricated using established techniques such as melt quenching, which enable good compositional control and structural homogeneity. In addition to their functional advantages, glasses are relatively inexpensive and easy to manufacture, making them practical candidates for large-scale shielding applications. In the present work, several critical design considerations are considered in the development of the proposed glass compositions. These include achieving high mass density and excellent transparency within the infrared spectrum to enhance shielding properties and maximize the interaction probability between photons and the glass. This high interaction rate results in a significant reduction in the energy of ionizing radiation, effectively blocking its transmission through the glass. Enhancing glass density is a key strategy for improving radiation shielding performance. In previous studies, we successfully utilized additives such as silver, bismuth, and zinc to develop selenium- and tellurium-rich ChGs with better shielding capabilities. ChGs, in particular, have emerged as promising candidates for X-rays and gamma radiation blocking.^14–17^ Tin is known to serve as a network former or an intermediate species in ChGs, often adopting tetrahedral coordination to surrounding chalcogen atoms. The addition of cadmium introduces a heavier divalent cation with a filled 4d^10^ electronic configuration and a relatively large ionic radius. Depending on its interaction with the host network, Cd may act as a modifier that disrupts Se–Se chains or as a cross-linking species forming Cd–Se structural units. Such incorporation is expected to influence atomic packing density, bond distribution, and overall network compactness. Structural compactification in multicomponent chalcogenide systems directly affects electron density distribution and interatomic spacing. These parameters govern the probability of photon interaction *via* photoelectric absorption and scattering, which are strongly dependent on atomic number (*Z*) and the electronic environment. Therefore, systematic compositional tuning provides an indirect but sensitive probe of structural evolution in these amorphous materials.

The efficiency of multicomponent chalcogenide glass systems depends on their composition and the intensity of the gamma radiation. The current investigation builds on this foundation by examining the radiation shielding properties of a specific chalcogenide glass system. As identified in previous studies, ternary and quaternary glasses with high chalcogen concentrations, particularly selenium, are promising candidates for radiation shielding applications. Additionally, chemical modifiers such as cadmium have been selected for their compatibility with other materials and potential to enhance specific shielding applications. The choice of these modifiers will be driven by considerations including improved safety, enhanced performance, environmental sustainability, cost-effectiveness, material availability, and the specific requirements of the intended radiation-shielding application. In the present work, SeTeSnCd system were synthesised to examine how progressive cadmium incorporation alters the structural and physical characteristics of the network. Particular attention is given to density variations, atomic packing considerations, and their correlation with photon-interaction behaviour. The objective is to elucidate how coordination modification in chalcogenide systems leads to measurable changes in the macroscopic attenuation response.

## 2. Experimental details

High-purity elements used in the present work were procured in sufficient quantities from a reputed supplier [Alfa Aesar, USA]. It was determined that each of these elements could be transformed into four distinct glassy samples through precision weighing, utilising a digital scale capable of detecting changes of 0.1 mg: (i) Se_78_Te_20_Sn_2_, (ii) Se_76_Te_20_Sn_2_Cd_2_, (iii) Se_74_Te_20_Sn_2_Cd_4_, and (iv) Se_72_Te_20_Sn_2_Cd_6_. The number of suffixes represents the proportions of atomic weights. In this study, ‘SeTeSn’ refers to the ternary system, while ‘SeTeSnCd’ denotes the quaternary system. The glassy systems, denoted Te_20_Sn_2_Cd_*x*_Se_78−*x*_ (with *x* ranging from 0 to 6), represent the investigated compositional variations. Subsequently, samples of the four distinct compositions were meticulously encased in uniform quartz tubes. Using a conventional sealing apparatus, these tubes were hermetically sealed under an inert atmosphere at 10^−6^ torr pressure. The highly evacuated tubes were then securely mounted on ceramic rods, facilitating their insertion into an electronic furnace for heating. This prolonged heating stage was crucial to ensure uniformity throughout the samples; accordingly, the sealed quartz ampoules were held at the target temperature for approximately 10–12 hours, with intermittent agitation to promote thorough mixing. Upon completion of the heating period, the molten samples were rapidly quenched by immersion in ice-cooled water, thereby enabling assessment of short-range ordering (SRO) characteristics within the concentration range.^18–20^ The sample ingots were prepared by breaking the quartz ampoules. Density measurements were performed using the Archimedes method with a high-precision density balance. The instrumental uncertainty was negligible compared with the observed compositional variations in density.

**Fig. 1:**
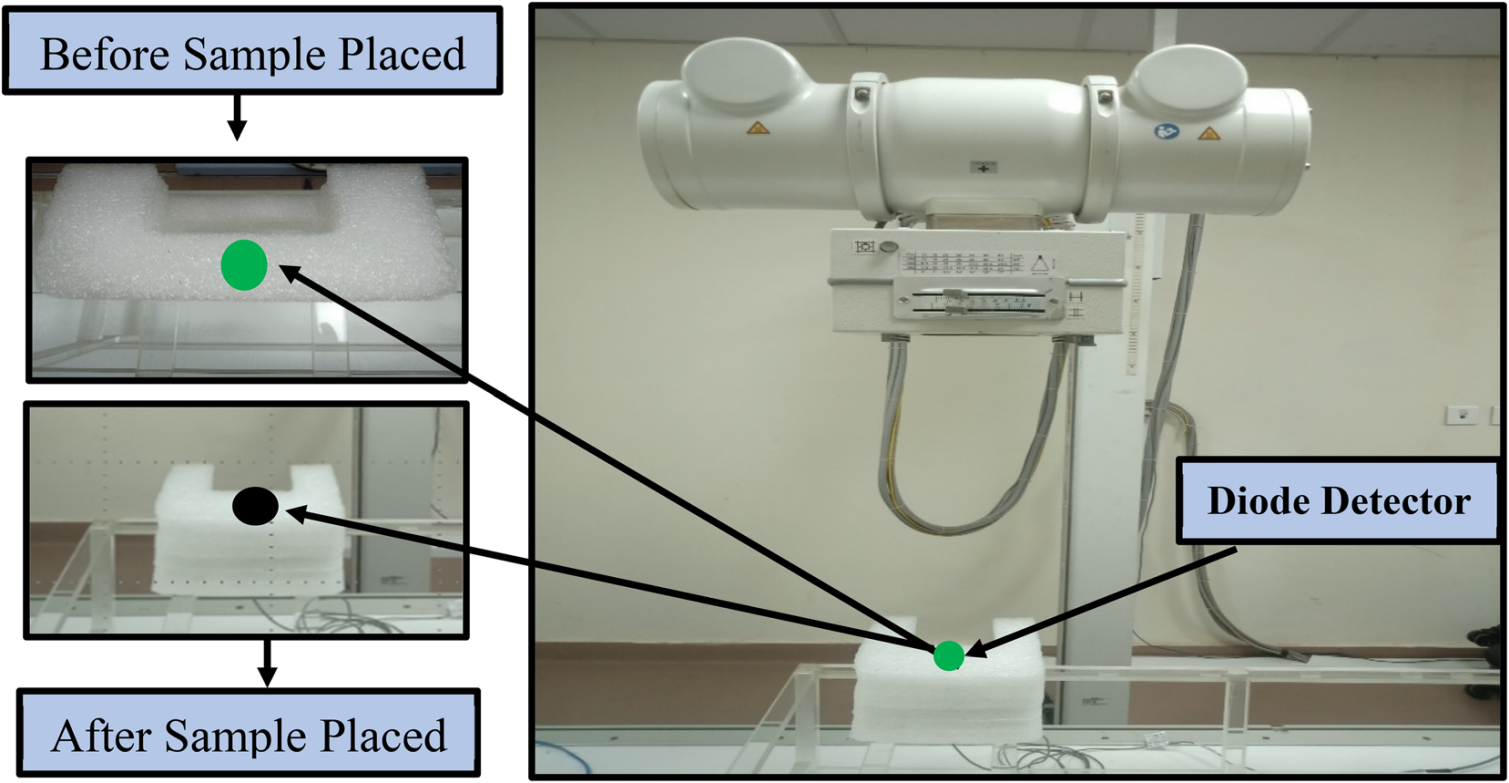
Experimental setup for irradiating 60 keV, 80 keV, and 100 keV of X-ray energies.

The amorphous phase of the samples was analyzed by X-ray diffraction (XRD) on a Panalytical Empyrean system, equipped with Cu K_α_ radiation (wavelength = 1.5406 Å). The measurements were conducted over a 10° to 90° scanning range, at 45 kV and 40 mA, with a scan step size of 0.0130°. The XRD characterisation utilised the same instrument's hybrid detector technology (PIXcel3D). The thermal behaviour of the multicomponent glasses was investigated using a Q20 model differential scanning calorimeter (DSC) from TA Instruments, USA. A consistent powder mass of 5 mg was used for each measurement, and the samples were heated at a controlled rate in the DSC. Heat flow variations were recorded at 15 K min^−1^ heating rates relative to an empty reference pan. The instrument provides a temperature accuracy of ±0.1 K, with a standard error of approximately 1 K for the observed results.^19,21–23^ Further, the composition of the Se_76_Te_20_Sn_2_Cd_2_ sample in pellet form was uniformly irradiated with X-rays at varying doses using a tungsten source in a high-intensity X-ray irradiation chamber. For this purpose, we plan to use the Siemens Polydoros-LX® (Siemens Healthcare GmbH, Germany) medical diagnostic X-ray tube equipped with a high-frequency generator (800 mA, 150 kV), as shown in Fig. 1. The X-ray tube blades were adjusted to produce a 5 × 5 cm^2^ field at a distance of 65 cm from the focal spot. Radiation from the X-ray tube was measured using a Stereotactic Field Diode Detector (IBA Dosimetry, GmbH, Germany) positioned at a focal spot-to-detector distance of 65 cm, with its detector chip oriented perpendicular to the central beam. The charge generated in the diode detector by X-ray interactions will be recorded using the Dose 1 Electrometer (Scanditronix Wellhofer, IBA Dosimetry GmbH, Germany), connected to the detector *via* a low-noise (≤15 fA leakage) triaxial cable. To measure the transmission through the developed samples, the detector was first exposed to an open field (without a sample in the beam). Subsequently, each sample was placed individually in the beam path, and the corresponding electrometer readings were recorded. For each measurement condition, five independent measurements (*n* = 5) were performed under identical experimental settings and averaged to obtain the final value. The results are presented as mean ± standard error (SE). The samples were tested at photon energies of 60, 80, and 100 keV, with exposure settings of 250 mA s and 630 ms, 250 mA s and 800 ms, and 90 mA s and 360 ms, respectively. These exposure parameters were selected to ensure that the leakage-to-main signal ratio remained below 0.1%.^24^ The ratio of transmitted readings through the sample to the corresponding open-field readings was used to determine the transmission values for each energy level investigated. The repeated incident-and-transmitted intensity measurements were subsequently used to calculate the experimental attenuation parameters, including the linear attenuation coefficient (LAC), transmission factor (TF), and radiation protection efficiency (RPE), using the Beer–Lambert law and related equations. Radiation attenuation measurements were performed for sample thicknesses ranging from 0.5 to 2.0 mm. In this manuscript, we report the XRD and DSC results for all prepared samples, along with the experimental radiation-shielding performance of the Se_76_Te_20_Sn_2_Cd_2_ chalcogenide glass system.

## 3. Computational methodology and theoretical framework

The XCOM application is a widely adopted web-based platform for accurately determining the mass attenuation coefficients (MAC) of materials under specified radiation conditions.^25,26^ Its popularity arises from its intuitive user interface, flexibility in defining material composition and photon energy ranges, and straightforward online accessibility, making it a reliable tool for radiation shielding analysis. A key feature of the program involves considering the material's elemental composition, a pivotal parameter in initiating the assessment. This elemental composition data is vital in computing the material's MAC, symbolised as (*µ*_m_). However, it's worth noting that XCOM, as a tool, is primarily designed for MAC determination and doesn't extend to calculating LAC or other pertinent shielding factors. In their latest study, Sakar *et al.* addressed this issue by introducing a novel online programme that can compute various shielding parameters. They christened their program Photon Shielding and Dosimetry (PSD) software, resolving the aforementioned challenges.^26^ This advancement represents a significant step forward in radiation shielding studies and is an invaluable tool available to registered users on the Phy-X platform. To effectively manage the data required for computing parameters (such as *Z*_eff_, MAC, HVL, and RPE) necessary for radiation shielding analysis in dosimetry, it provides an intuitive interface.^25–27^

The average track length of incoming gamma photons was simulated using the Monte Carlo N-Particle Transport Code (MCNP-5), a valuable non-destructive tool. Based on the simulated average track length, several key shielding factors were calculated as follows:^27–32^1
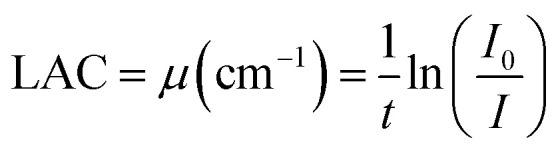
2
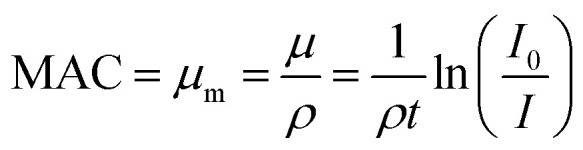
3
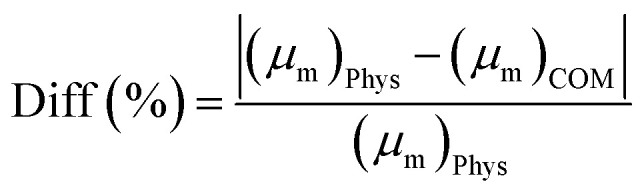
4
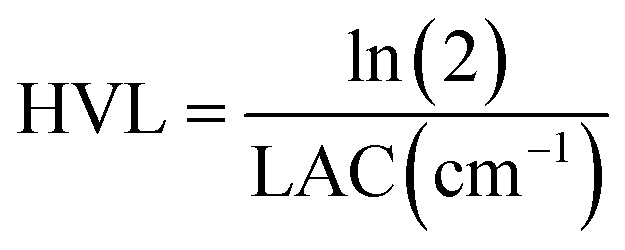
5
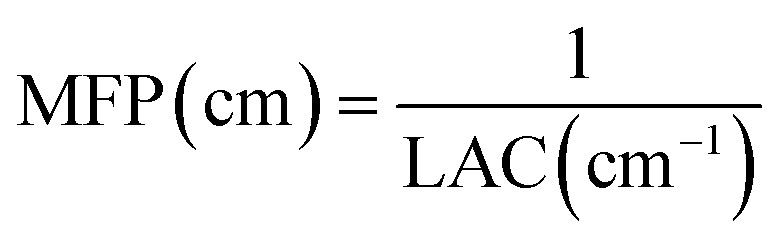
6
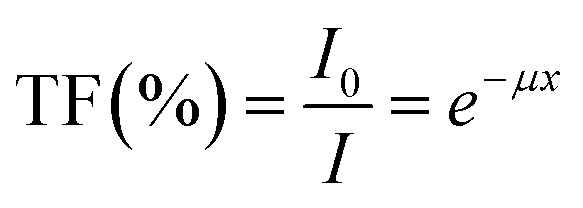
7
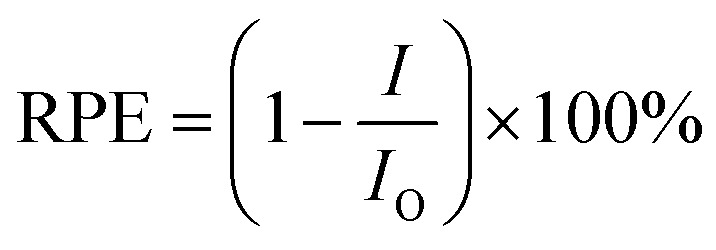


In the above equations, LAC, MAC, HVL, MFP, TF, and RPE represent the corresponding shielding parameters for a sample of thickness *t*.^33–38^

## 4. Results and discussion

**Fig. 2:**
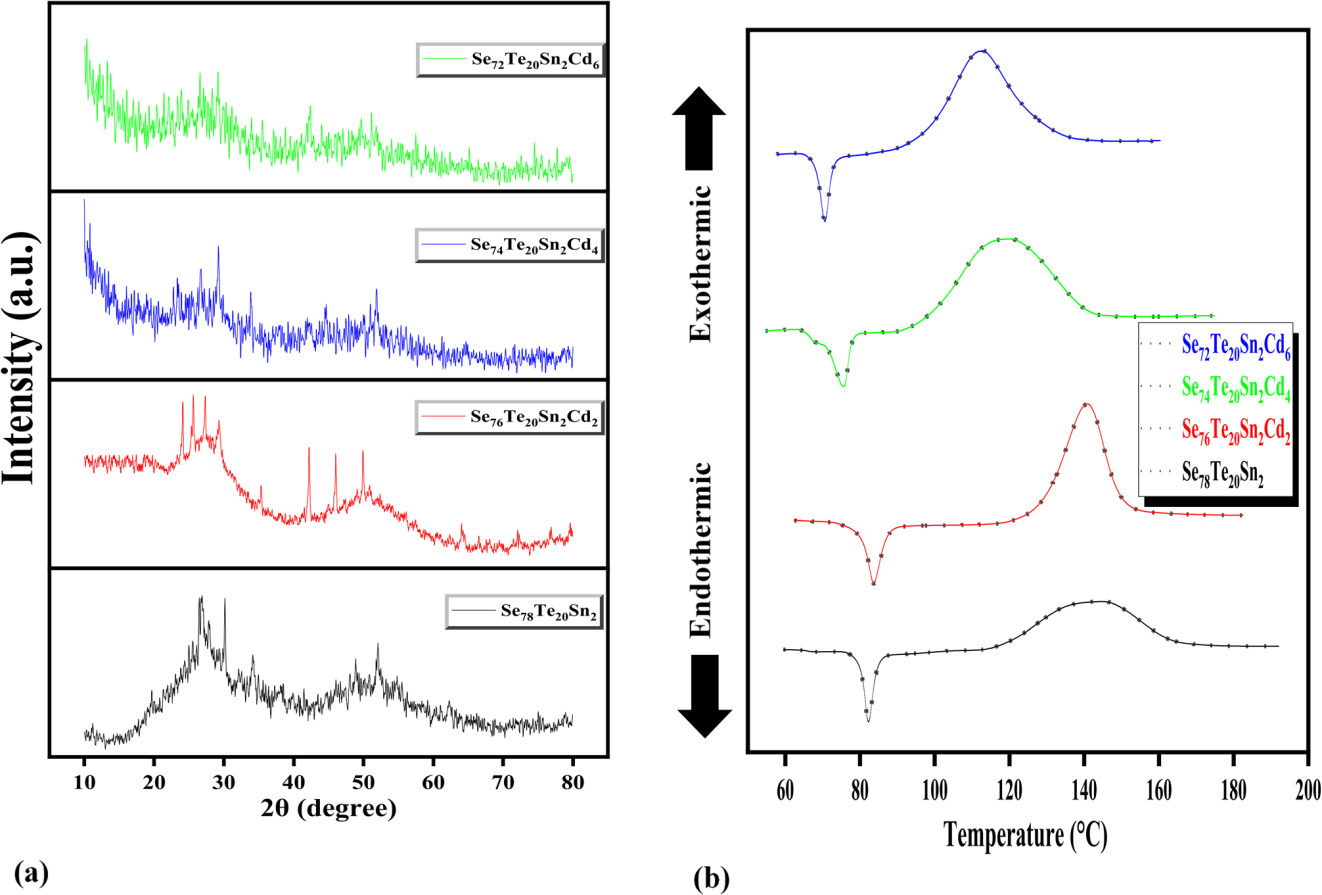
(a) XRD pattern of the as-prepared SeTeSn and SeTeSnCd sample, (b) DSC scan of the SeTeSn and SeTeSnCd sample.

Fig. 2(a) shows the XRD patterns of the as-prepared Se_78−*x*_Te_20_Sn_2_Cd_*x*_ (*x* = 0, 2, 4, 6) samples. The undoped STS sample exhibits a broad diffuse halo without intense Bragg reflections, confirming the predominantly amorphous/glassy nature of the alloy. Such broad diffraction humps are typical of chalcogenide glasses, where the absence of long-range periodicity gives rise to diffuse scattering rather than sharp crystalline peaks. The broad features observed in the low- and mid-angle regions may be assigned to the first- and second-sharp diffraction peaks, which are commonly associated with short- and intermediate-range ordering in amorphous chalcogenide networks. The FSDP is widely considered a structural signature of intermediate-range order in chalcogenide glasses.^18–20^

With Cd incorporation, the overall amorphous background is retained; however, weak diffraction features become visible, especially for the Se_76_Te_20_Sn_2_Cd_2_ sample. These weak peaks are superimposed on the broad amorphous halo and therefore do not indicate complete crystallization. Instead, they suggest the onset of localized ordering or the formation of small nanocrystalline domains within the amorphous matrix. This effect may arise from Cd-induced structural rearrangement, where Cd modifies the SeTeSn network by forming CdSe/CdTe related bonding units and increasing local structural heterogeneity. For higher Cd contents, namely Se_74_Te_20_Sn_2_Cd_4_ and Se_72_Te_20_Sn_2_Cd_6_, the diffraction patterns again remain largely dominated by broad halos with only weak features, indicating that the glassy character is mostly preserved. Therefore, the XRD results confirm that all samples are predominantly amorphous, while Cd addition promotes limited intermediate-range ordering and, in the Cd_2_ composition, slight incipient crystallization.^18–20^

Fig. 2(b) presents the DSC curves of the undoped and Cd-doped SeTeSn glassy samples recorded at a heating rate of 15 K min^−1^. Each thermogram shows a distinct endothermic event followed by an exothermic crystallization peak. The first endothermic deviation corresponds to the glass transition temperature (*T*_g_), where the rigid glassy network transforms into a supercooled liquid state. The subsequent exothermic peak corresponds to crystallization, (*T*_c_), caused by thermally activated nucleation and growth of crystalline phases within the amorphous matrix. DSC is commonly used to identify (*T*_g_), crystallization onset/peak temperature, and melting-related events in chalcogenide glasses.^19,21^ The presence of well-defined glass transition and crystallization features confirms that the as-prepared samples are glassy and undergo thermally induced crystallization during heating. The sharpness of the glass transition and crystallization peaks also indicates reasonable compositional homogeneity of the prepared alloys. Upon Cd addition, noticeable changes occur in the position and intensity of the crystallization peaks, showing that Cd affects the crystallization kinetics of the SeTeSn network. The shift and modification of the exothermic peaks suggest changes in network rigidity, atomic mobility, and nucleation tendency. In particular, the Cd-containing samples show more pronounced crystallization behavior than the undoped glass, which is consistent with the weak ordering features observed in the XRD patterns. The combined XRD and DSC results therefore indicate that Cd doping does not destroy the glassy nature of the SeTeSn matrix, but it significantly modifies the local structure and thermal crystallization response. The XRD patterns confirm predominantly amorphous behavior with weak Cd-induced ordering, while the DSC curves demonstrate clear glass transition and crystallization events. This correlation supports the view that Cd incorporation tunes the intermediate-range structure and thermal stability of the STSC glassy system, which is important for evaluating its suitability for phase-change and related optoelectronic applications.^18–22^

**Fig. 3:**
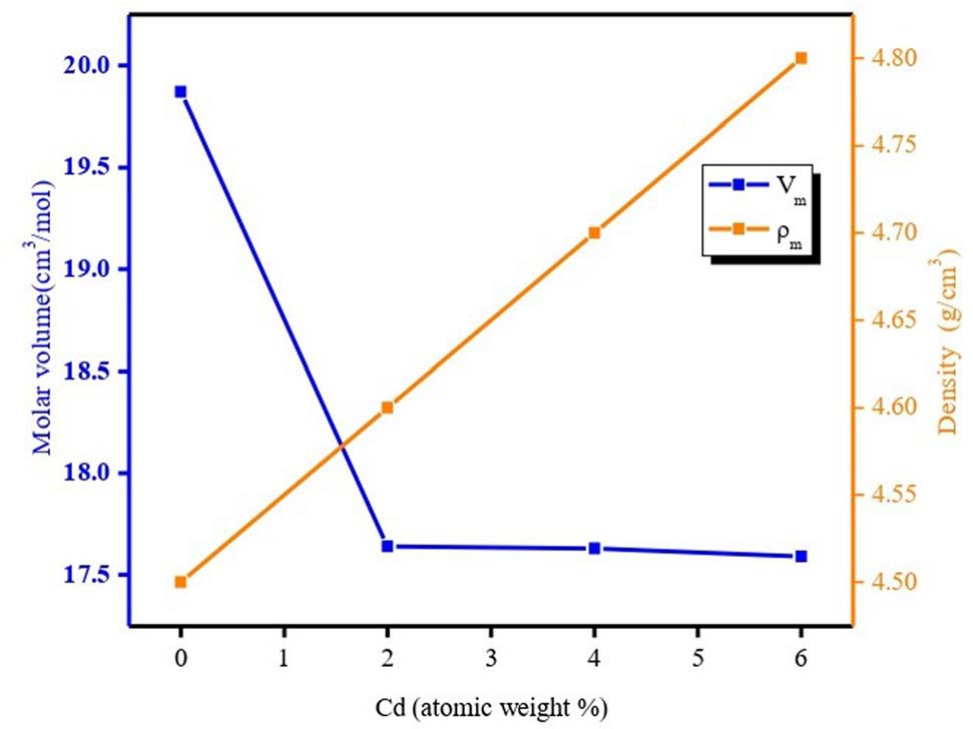
Composition-dependent variation in density and *V*_m_ for SeTeSn and SeTeSnCd glass samples as a function of Cd content (0 ≤ *x* ≤ 6).

**Fig. 4:**
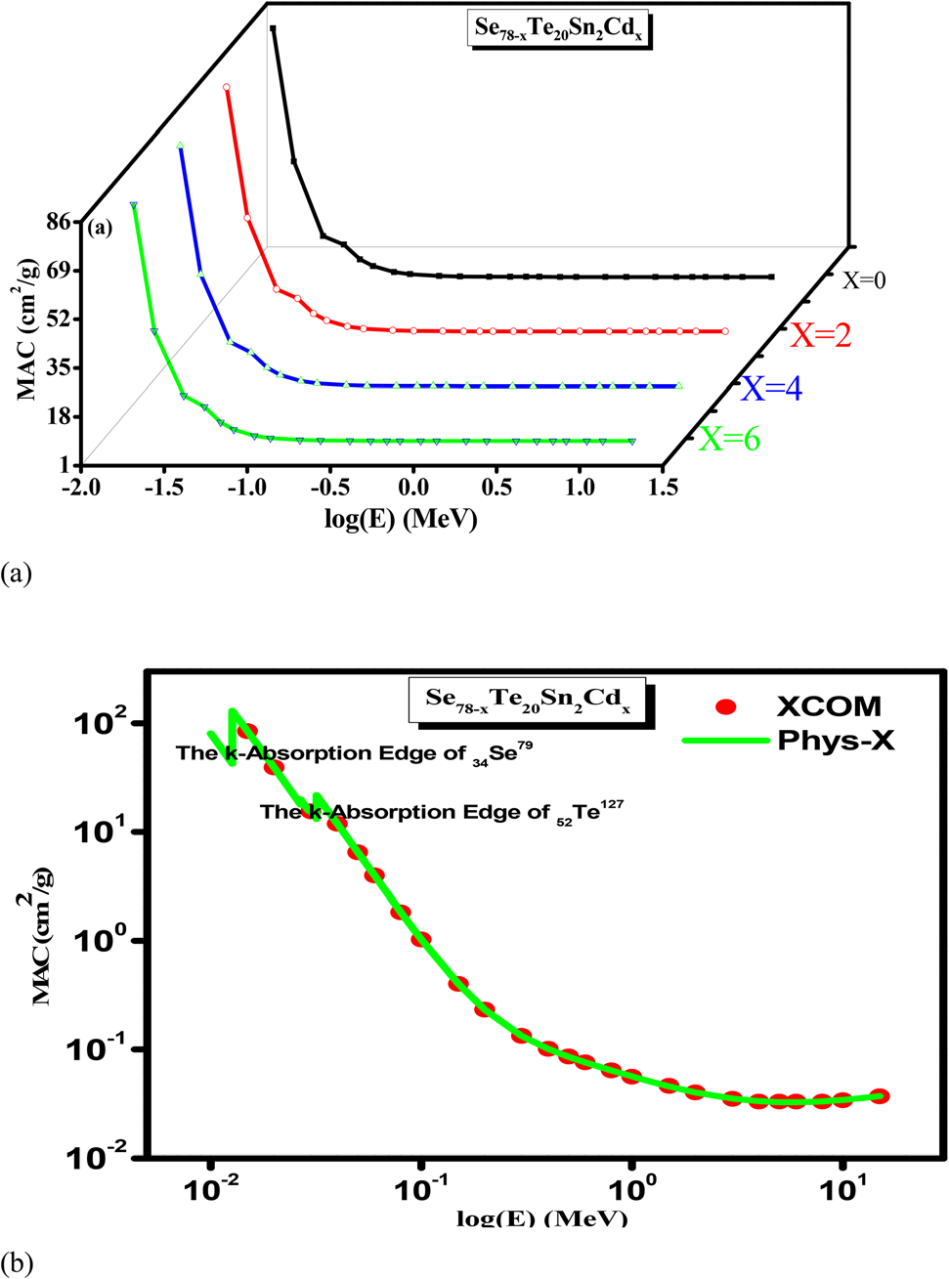
(a) MAC plotted as a function of log photon energy (*E*) in the range of 0.015–15 MeV for the investigated glass compositions. (b) MAC results for the Cd-4 sample (*x* = 4), computed using the Phys-X online platform. K-absorption edges corresponding to isotopes _34_Se^79^ and _52_Te^127^ are indicated. Theoretical results obtained from deterministic calculations are shown without error bars.

**Fig. 5:**
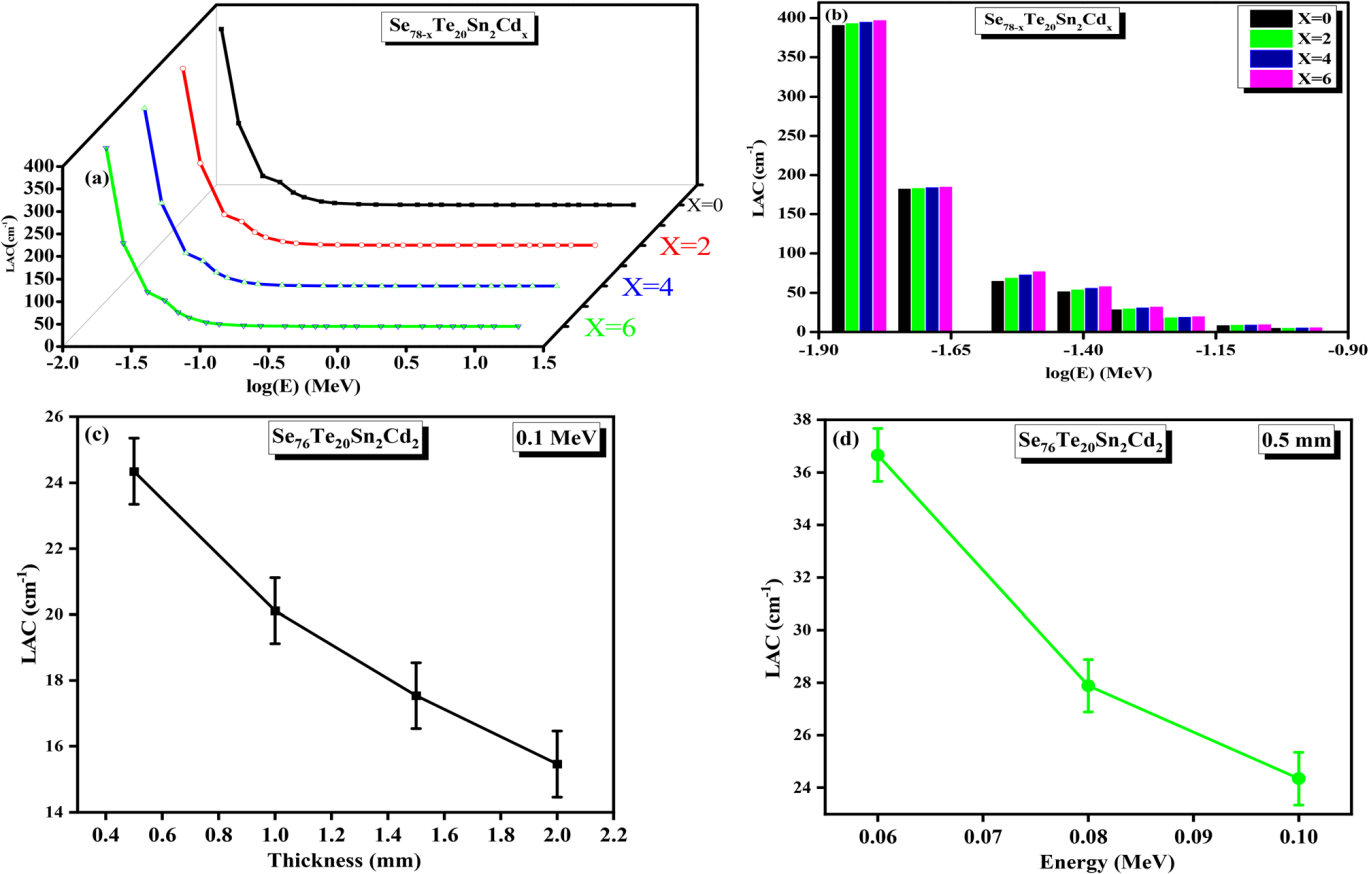
Plots of (a) the LAC as a function of incoming photon energy and (b) the LAC values at selected incoming photon energies for the SeTeSn and SeTeSnCd sample (c) thickness-dependent experimental LAC at particular energy 0.1 MeV (d) energy-dependent experimental LAC at a particular thickness of 0.5 mm. Error bars represent the standard error of the mean calculated from five repeated measurements (*n* = 5).

Moreover, Table 1 presents the experimental physical properties of the samples. The samples’ density increased with the cadmium additive concentration. Specifically, when the cadmium content increased, the density rose significantly from 4.50 to 4.8 g cm^−3^. A density balance meter (Contech Electronic Balance, India; model: CA-44) was used for these measurements. This relationship is depicted in Fig. 3, which also shows the least-squares linear fit to the obtained densities. The observed increase in density with cadmium incorporation suggests progressive structural reorganisation within the amorphous network. In Se-rich glasses, the structure is typically dominated by chain-like Se units and Se–Te heteropolar bonds. The introduction of Cd^2+^ is expected to alter this arrangement by forming Cd–Se linkages that may serve as cross-linking sites within the network. Considering ionic size and valence requirements, cadmium is likely to adopt a higher coordination environment with surrounding chalcogen atoms. The replacement of lighter Se atoms by heavier Cd species increases mass density while simultaneously reducing free volume. This process enhances atomic packing efficiency and decreases interatomic separation. The structural densification is therefore not merely compositional but also reflects the evolution of coordination within the glass matrix. Such reorganisation increases the overall effective electron density (*N*_eff_) of the system and modifies the local bonding distribution, both of which play a central role in the probability of photon–matter interaction. The molar volume (*V*_m_) shows a non-linear decrease from 19.87 to 17.59 cm^3^ mol^−1^ with increasing Cd content, while the density (*ρ*) increases nearly monotonically from 4.50 to 4.80 g cm^−3^. Thus, *V*_m_ and *ρ* change by approximately 2.28 cm^3^ mol^−1^ and 0.30 g cm^−3^, respectively. The incorporation of heavier Cd^2+^ ions replaces lighter selenium atoms and likely promotes additional cross-linking through Cd–Se interactions. The concurrent decrease in *V*_m_ suggests reduced free volume and enhanced atomic packing. Such densification indicates that cadmium incorporation induces reorganisation of short-range order rather than simple compositional substitution.^39,40^ These values are associated with higher elastic moduli in ChGs and related network glasses.^25^ The MAC determines a material's ability to attenuate photon radiation. In the present work, the MAC values exhibit significant variations across different photon energies and cadmium concentrations, as shown in Fig. 4. The alloy demonstrates higher MAC values at lower photon energies, specifically at 0.015 and 0.02 MeV. As the cadmium content increases, there is a corresponding increase in MAC values, ranging from 82.622 to 86.794 cm^2^ g^−1^ at 0.015 MeV and from 39.08 to 40.409 cm^2^ g^−1^ at 0.02 MeV. This trend indicates that higher cadmium concentrations enhance the alloy's ability to attenuate low-energy photons. However, as the photon energy increases from 0.03 to 0.2 MeV, the MAC values decrease significantly. For instance, in the alloy without cadmium (*x* = 0), the MAC decreases from 14.328 cm^2^ g^−1^ at 0.03 MeV to 0.226 cm^2^ g^−1^ at 0.2 MeV. Similarly, for the alloy with the highest cadmium content (*x* = 6), the MAC decreases from 15.96 cm^2^ g^−1^ at 0.03 MeV to 0.234 cm^2^ g^−1^ at 0.2 MeV. Furthermore, the sample with the highest cadmium concentration exhibits the lowest MAC values at medium photon energies. As the photon energy increases, the MAC values for all samples converge, becoming nearly identical for energies above 0.15 MeV. This convergence suggests that at higher photon energies, the alloy's composition has a diminishing effect on MAC, leading to similar attenuation characteristics across different cadmium concentrations. These observations align with general trends in photon–matter interactions. The variation of the MAC with photon energy reflects the transition between dominant photon–matter interaction mechanisms. In the low-energy region (below ∼0.1 MeV), the MAC values are significantly higher and increase with cadmium content. This behaviour arises from the dominance of the photoelectric effect, whose probability strongly depends on *Z* (approximately proportional to *Z*^4^–*Z*^5^). The incorporation of Cd, possessing a higher *Z* than selenium, increases the effective atomic number (*Z*_eff_) of the glass system. As a result, the probability of photoelectric absorption rises markedly with increasing Cd concentration. In this regime, even small compositional changes substantially influence attenuation behaviour. As photon energy increases into the intermediate region, the MAC values decrease rapidly for all compositions. In this range, Compton scattering becomes the dominant interaction mechanism. Unlike the photoelectric effect, Compton scattering depends primarily on electron density rather than strongly on *Z*. Since the total electron density differences among the compositions are comparatively modest, the influence of cadmium becomes less pronounced. Consequently, the MAC values across different compositions begin to converge. At higher photon energies, the MAC values decrease further and eventually converge across all samples. In this region, pair production and high-energy scattering processes dominate. These mechanisms are less sensitive to moderate variations in composition within this *Z* range. Therefore, although cadmium incorporation increases *ρ* and *Z*_eff_, its impact on attenuation becomes progressively weaker at higher energies. The diminishing compositional dependence of MAC in this region reflects the reduced sensitivity of high-energy photon interactions to local structural modifications. This behaviour is primarily attributed to the strong dependence of the photoelectric effect on atomic number, where the absorption cross-section scales as *Z*^4^–*Z*^5^.^33,35^ The substitution of selenium (*Z* = 34) by cadmium (*Z* = 48) increases the effective atomic number (*Z*_eff_) of the glass system, thereby enhancing the probability of photoelectric absorption. Additionally, the structural compactification induced by Cd incorporation – evidenced by increased density (4.50 → 4.80 g cm^−3^) and reduced molar volume (19.87 → 17.59 cm^3^ mol^−1^) – increases the electron density (*N*_eff_) per unit volume, further contributing to the observed MAC enhancement.^15^ As photon energy increases beyond 0.1 MeV, the MAC values decrease for all compositions, and the relative enhancement due to cadmium becomes progressively smaller. This trend reflects the transition from photoelectric dominance to Compton scattering, which depends primarily on total electron density rather than atomic number. Consequently, at energies above 1.0 MeV, the MAC values across different compositions converge.^38,39^ Furthermore, we compared the MACs of our prepared samples at 0.02 MeV and 10 MeV with those reported for other materials, as shown in Table 2. The results indicate that our samples exhibit superior radiation attenuation, highlighting their effectiveness as promising shielding materials across a broad range of photon energies.^40–45^ Moreover, Fig. 5(a) and (b) illustrate the fluctuations in the LAC as photon energy increases. The LAC is a fundamental parameter that quantifies the probability of photon interactions per unit distance travelled in a material. Its behaviour across varying photon energies and material compositions provides key insights into the material's radiation shielding effectiveness. In the photon energy range of 0.15 to 15 MeV, the LAC values for the studied material range from approximately 396.50 cm^−1^ to 0.179 cm^−1^. This trend mirrors that of the MAC, underscoring their intrinsic relationship. Both coefficients are influenced by factors such as photon energy, material density, and *Z*. As photon energy increases, the probability of interaction per unit distance generally decreases, leading to lower LAC values. The novel Se–Te–Sn–Cd glassy alloys under investigation exhibit higher LACs across all energy levels than standard commercial materials such as RS 360, RS 253G18, and Types A, B, and C, as shown in Table 3. The observed ranking in shielding effectiveness is Cd6 > Cd4 > Cd2 > RS 360 > RS 253G18 > Type A > Type B > Type C, indicating that the Cd-doped glassy alloys exhibit greater radiation attenuation properties.^45–52^ This enhanced performance can be attributed to the specific composition and structure of the SeTeSnCd alloys, which facilitate more effective photon interactions. The incorporation of cadmium, with its high *Z*, increases the probability of photon interactions, such as photoelectric absorption and Compton scattering, thereby increasing the attenuation coefficients. Additionally, the glassy matrix provides a dense, amorphous structure, further enhancing the material's ability to attenuate radiation. In contrast, standard materials such as RS 360 and RS 253G18, while effective, may not provide the same level of attenuation due to differences in composition and *ρ*. The better performance of the Se–Te–Sn–Cd alloys suggests their potential for applications in environments requiring enhanced radiation shielding, offering a promising alternative to conventional materials. In Fig. 5(c), when examining the dependence of LAC on material thickness at a photon energy of 0.1 MeV, an inverse relationship is observed. As the thickness increases, the LAC decreases. The LAC is an intrinsic property of the material and represents the probability of photon interaction per unit path length. In principle, LAC is independent of sample thickness, as it is determined by composition, *ρ*, and photon energy. The apparent variation observed in experimentally derived values with increasing thickness arises from attenuation fitting over finite thickness ranges and measurement sensitivity at low transmitted intensities. Therefore, thickness primarily influences transmitted intensity rather than the intrinsic attenuation coefficient.^53–58^

**Table 1:** The chemical composition *x* of cadmium, density, and molar volume for the prepared Te_20_Sn_2_Cd_*x*_Se_78−*x*_ (0 ≤ *x* ≤ 6) systems

Cd-*x*	Chemical compositions (at. wt%)				Density (g cm^−3^)	*V*_m_ (cm^3^ mol^−1^)
	Se	Te	Sn	Cd		
Cd-0	78	20	2	0	4.5	19.87
Cd-2	76	20	2	2	4.6	17.64
Cd-4	74	20	2	4	4.7	17.63
Cd-6	72	20	2	6	4.8	17.59

**Table 2:** Comparison of MAC parameters for the present samples with those of other studied samples

Samples	MAC (MeV)		Ref.
	0.02	10	
Se_78_Te_20_Sn_2_	40.22	0.0340	Present work
Se_76_Te_20_Sn_2_Cd_2_	39.74	0.0341	
Se_74_Te_20_Sn_2_Cd_4_	39.08	0.0342	
Se_72_Te_20_Sn_2_Cd_6_	38.44	0.0344	
BPLM 5	11.57	0.023	40–44
66B_2_O_3_–5Al_2_ O_3_–29Na_2_O	1.074	0.020	
5Bi_2_O_3_–61B_2_O_3_–5Al_2_O_3_–29Na_2_O	5.059	0.022	
10Bi_2_O_3_–56B_2_O_3_–5Al_2_O_3_–29Na_2_O	9.043	0.023	
0PbO–30SiO_2_–46.67B_2_O_3_–23.33Na_2_O	1.386	0.023	
5PbO–25SiO_2_–46.67B_2_O_3_–23.33Na_2_O	5.167	0.021	
10PbO–20SiO_2_–46.67B_2_O_3_–23.33Na_2_O	8.952	0.024	
49.46SiO_2_–26.38Na_2_O–23.08CaO–1.07P_2_O_5_	3.982	0.024	
47.84SiO_2_–26.67Na_2_O–23.33CaO–2.16P_2_O_5_	3.985	0.023	
44.47SiO_2_–27.26Na_2_O–23.85CaO–4.42P_2_O_5_	4.057	0.024	
40.96SiO_2_-27.87Na_2_O–24.39CaO–6.78P_2_O_5_	4.113	0.024	
37.28SiO_2_–28.52Na_2_O–24.95CaO–9.25P_2_O_5_	4.061	0.024	
48.98SiO_2_–26.67Na_2_O–23.33CaO–1.02P_2_O_5_	3.983	0.023	
43.66SiO_2_–28.12Na_2_O–24.60CaO–3.62P_2_O_5_	4.1	0.024	
38.14SiO_2_–29.62Na_2_O–25.91CaO–6.33P_2_O_5_	4.19	0.022	
40.71SiO_2_–28.91Na_2_O–25.31CaO–5.07P_2_O_5_	4.131	0.022	
75SiO_2_–15Na_2_O–10CaO	3.081	0.0212	45
74SiO_2_–15Na_2_O–10CaO–ZrO_2_	4.118	0.0215	
72SiO_2_–15Na_2_O–10CaO–3ZrO_2_	6.128	0.0219	
70SiO_2_–15Na_2_O–10CaO–5ZrO_2_	8.058	0.0224	
68SiO_2_–15Na_2_O–10CaO–7ZrO_2_	9.912	0.0229	

**Table 3:** The theoretical LAC value of the present sample with commercial glasses

Energy (MeV)	Cd_0_	Cd_2_	Cd_4_	Cd_6_	Type A glass	Type B glass	Type C glass	RS 253-G18	RS-360
0.06	27.94	19.79	20.35	20.95	0.87	0.88	0.52	2.09	12.01
0.1	4.46	5.16	5.30	5.45	0.47	0.47	0.31	0.78	13.25
0.5	0.38	0.44	0.44	0.45	0.22	0.22	0.15	0.22	0.39
1	0.25	0.29	0.29	0.29	0.16	0.16	0.11	0.16	0.18
5	0.15	0.17	0.17	0.17	0.07	0.07	0.05	0.08	0.11
10	0.153	0.17	0.18	0.18	0.06	0.06	0.04	0.06	0.12
15	0.16	0.19	0.19	0.19	0.06	0.06	0.04	0.06	0.14

**Fig. 6:**
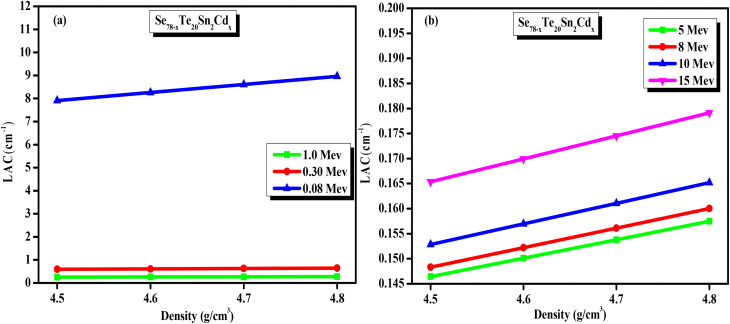
Plots showing the variation of LAC with density at (a) low energy range and (b) high energy range for the SeTeSn and SeTeSnCd study. Theoretical results obtained from deterministic calculations are shown without error bars.

Conversely, in Fig. 5(d), the LAC decreases with increasing photon energy at a constant thickness. This behaviour aligns with the general understanding that higher-energy photons have a lower probability of interacting with the material, resulting in lower attenuation coefficients. Furthermore, the dependence of the LAC on material *ρ*, as shown in Fig. 6, indicates that, particularly at elevated photon energies, denser samples exhibit higher LAC values, highlighting the role of material compactness in enhancing photon attenuation. This is consistent with theoretical expectations, as denser materials present more atoms per unit volume, increasing the likelihood of photon interactions. However, at lower photon energies, the LAC remains relatively constant with increasing *ρ*, suggesting that other factors, such as *Z* and specific interaction mechanisms, play more significant roles in attenuation at these energies. The dependence of LAC on *ρ* reflects the increase in the number of interaction centres per unit volume. At low photon energies, where photoelectric absorption dominates, LAC increases markedly with both *Z* and *ρ*. The progressive densification induced by cadmium incorporation enhances the probability of photon interaction by reducing interatomic spacing and increasing electron density per unit volume. At higher photon energies, the sensitivity of *µ* to *ρ* becomes less pronounced because Compton scattering depends primarily on total electron density rather than strongly on *Z*. Consequently, compositional differences exert a weaker influence in this regime.

**Fig. 7:**
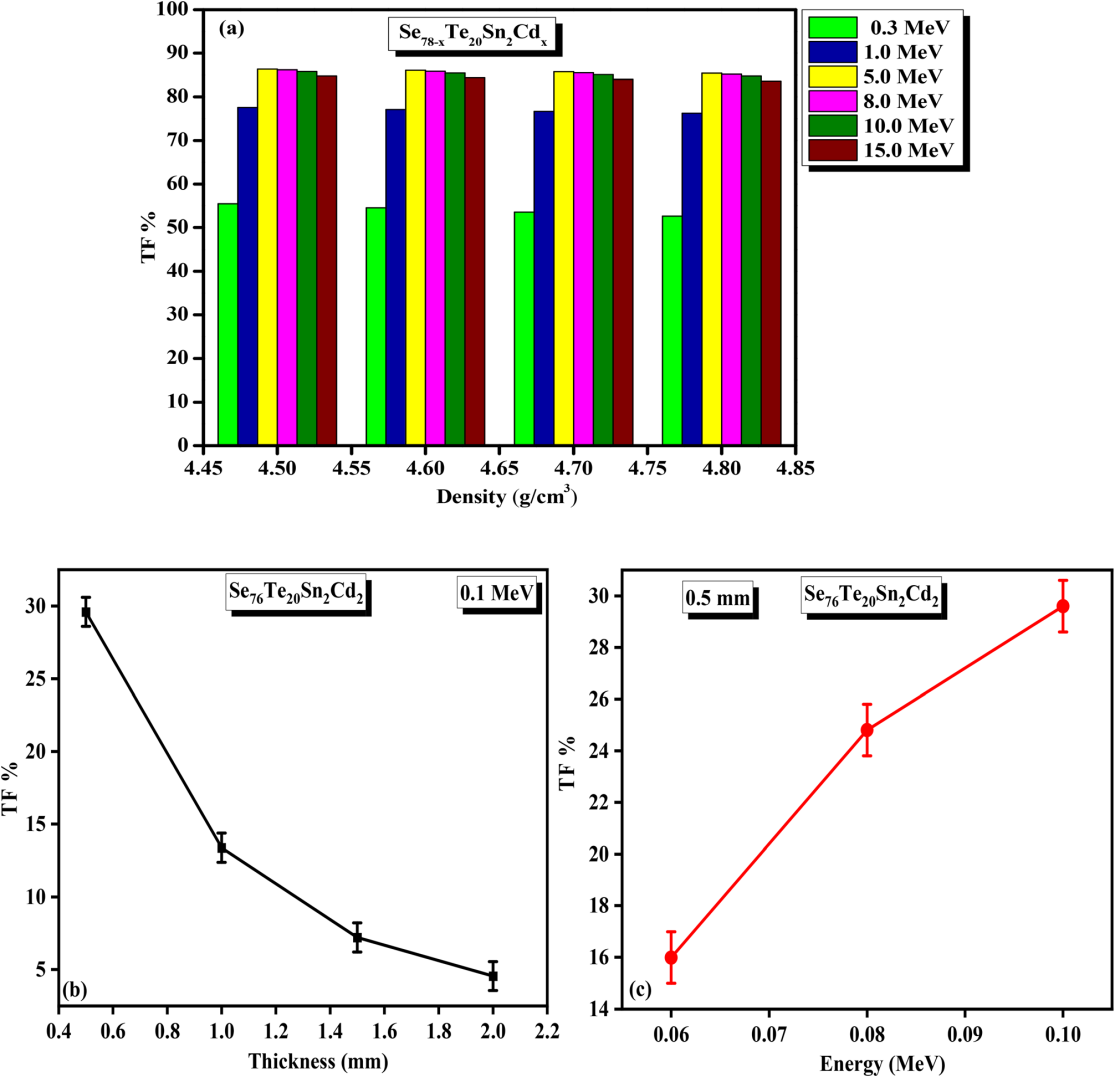
(a) The TF% compositional SeTeSn and SeTeSnCd sample (b) thickness-dependent experimental TF% at a particular energy of 0.1 MeV (c) energy-dependent experimental TF% at a particular thickness of 0.5 mm. Error bars represent the standard error of the mean calculated from five repeated measurements (*n* = 5).

Fig. 7(a) illustrates that the TF% of a material, which quantifies the fraction of incident radiation that passes through without attenuation, is influenced by several factors, including photon energy, material thickness, and *ρ*. As photon energy increases, the TF% generally increases, indicating that higher-energy photons are less likely to be attenuated by the material. Higher-energy photons have a lower probability of interacting with atoms in the shielding material, leading to reduced attenuation. However, at photon energies exceeding 5.0 MeV, a decrease in TF% has been observed, indicating increased attenuation. *ρ* also significantly impacts the TF%. Materials with higher *ρ* have atoms more closely packed together, increasing the probability of photon interactions and thus enhancing attenuation. Consequently, higher-*ρ* materials typically exhibit lower TF% values, indicating better shielding effectiveness. This behaviour can be attributed to the increased likelihood of pair-production interactions at higher photon energies, which contribute to greater attenuation.^24^ We also compared the TF% of our samples at different photon energies with those of commercial shielding materials, as shown in Table 4. The significantly lower TF% observed in our samples indicates their excellent radiation attenuation capability and overall shielding effectiveness. Fig. 7(b) shows that material thickness plays a key role in shielding effectiveness. As thickness increases, TF% decreases, indicating that a thicker material attenuates more incident radiation. This relationship arises because photon interactions increase as photons traverse a thicker medium, leading to greater attenuation. This inverse relationship between thickness and TF% is well documented in radiation-shielding studies. The material's thickness plays a key role in its shielding effectiveness. As thickness increases, TF% decreases, indicating that a thicker material attenuates more incident radiation. This relationship arises from the increased photon interactions as photons traverse a thicker medium, leading to greater attenuation. This inverse relationship between thickness and TF% is well documented in radiation-shielding studies. Fig. 7(c) shows the energy dependence at a constant material thickness; the TF% generally increases with photon energy. This trend arises because higher-energy photons have a lower probability of interacting with the material's atoms, leading to reduced attenuation. Consequently, as photon energy rises, more photons can traverse the material without being absorbed or scattered, resulting in a higher TF%. This behaviour is well documented in studies of the gamma-ray attenuation properties of common shielding materials.

**Table 4:** Theoretical value of TF% of the present sample with commercial glasses

Energy (MeV)	Cd_0_	Cd_2_	Cd_4_	Cd_6_	Type A glass	Type B glass	Type C glass	RS 253-G18	RS-360
0.06	0.00	0.00	0.00	0.00	41.99	41.64	59.39	12.33	0.00
0.1	1.16	0.57	0.50	0.43	62.41	62.29	73.24	45.82	0.00
0.5	68.20	64.67	64.37	64.01	80.46	80.45	85.87	79.96	67.38
1	77.59	74.95	74.76	74.53	85.38	85.38	89.51	85.18	83.81
5	86.38	84.64	84.49	84.32	92.88	92.87	95.06	92.77	90.01
10	85.82	84.00	83.82	83.61	94.03	94.02	95.99	93.93	88.58
15	84.76	82.79	82.59	82.36	94.28	94.27	96.23	94.17	87.16

**Fig. 8:**
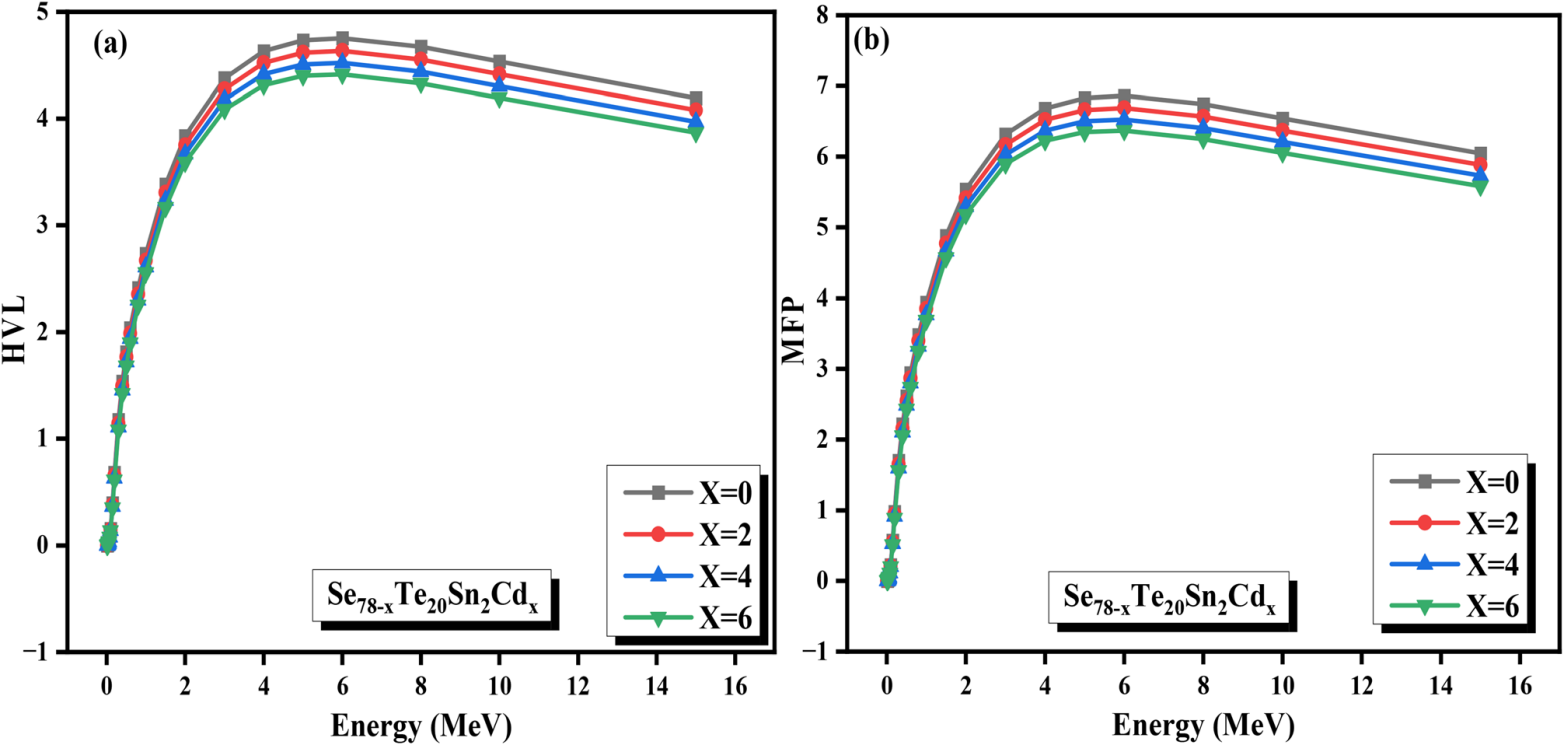
Variation of (a) HVL and (b) MFP with respect to gamma photon energy for the SeTeSn and SeTeSnCd glassy matrix. Theoretical results obtained from deterministic calculations are shown without error bars.

Fig. 8 presents the variation of the HVL and MFP as a function of photon energy for the investigated glass compositions. Both HVL and MFP are inversely related to the LAC and, therefore, provide an indirect measure of photon interaction probability within the material. Lower HVL and MFP values indicate stronger attenuation and higher interaction probability. In the low-energy region, where the photoelectric effect is dominant, HVL and MFP values are relatively small and show noticeable dependence on cadmium concentration. The incorporation of cadmium increases the *Z*_eff_ and enhances structural compactness by increasing *ρ* and reducing free volume. These structural modifications increase the probability of photon absorption, thereby reducing the HVL and MFP values for Cd-rich compositions. As photon energy increases into the intermediate region, HVL and MFP increase for all compositions. This behaviour reflects the transition from photoelectric absorption to Compton scattering, which is less sensitive to *Z* and more dependent on overall electron density. Consequently, although cadmium incorporation continues to contribute to attenuation through increased *ρ*, the relative differences among compositions become smaller in this energy range.

**Fig. 9:**
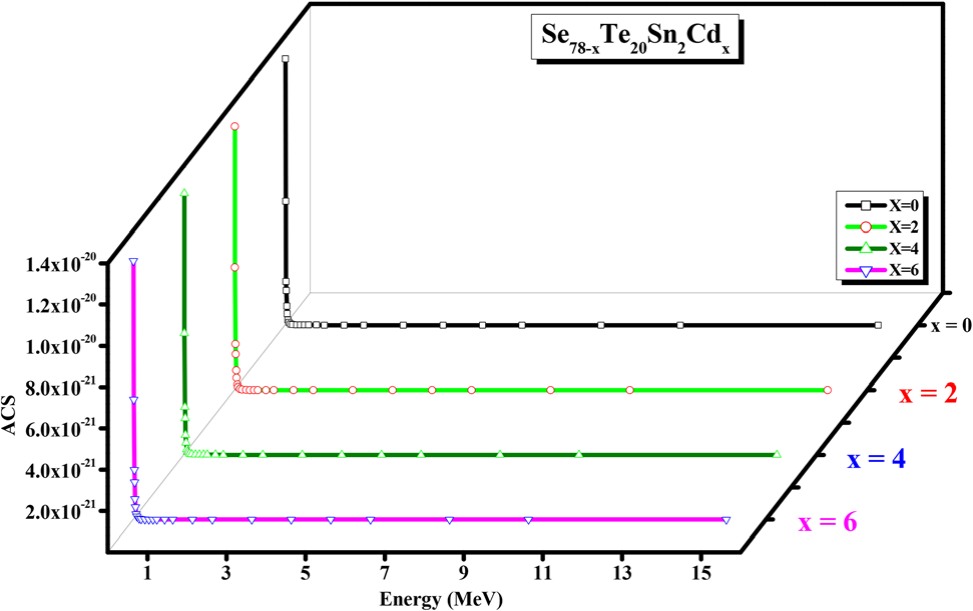
Variation of total ACS as a function of photon energy in the range of 0.15–15 MeV for the SeTeSn and SeTeSnCd glass system.

**Fig. 10:**
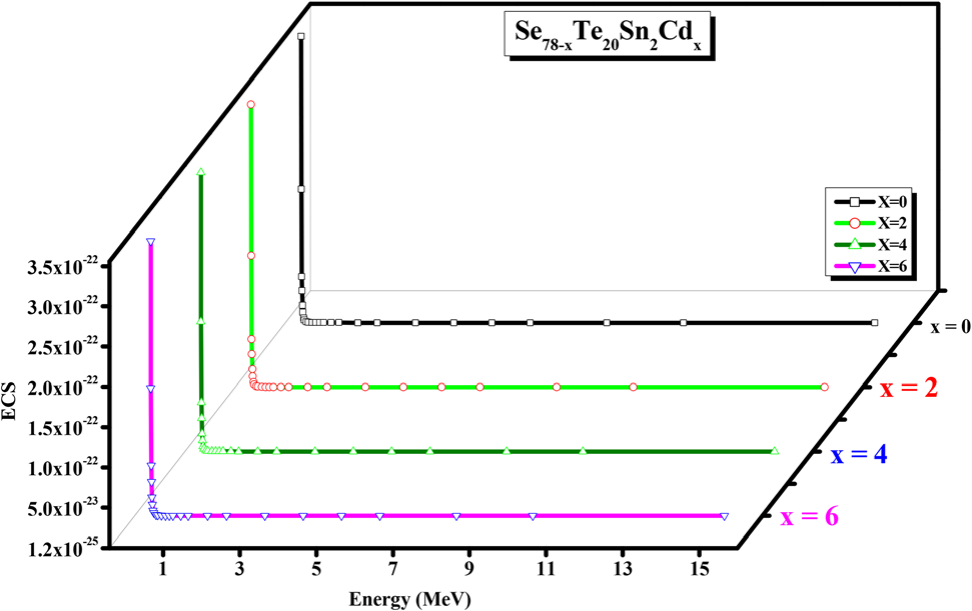
Photon energy dependence of the total electronic cross-section (ECS) in the range of 0.15–15 MeV for the SeTeSn and SeTeSnCd glass samples. Theoretical results obtained from deterministic calculations are shown without error bars.

**Fig. 11:**
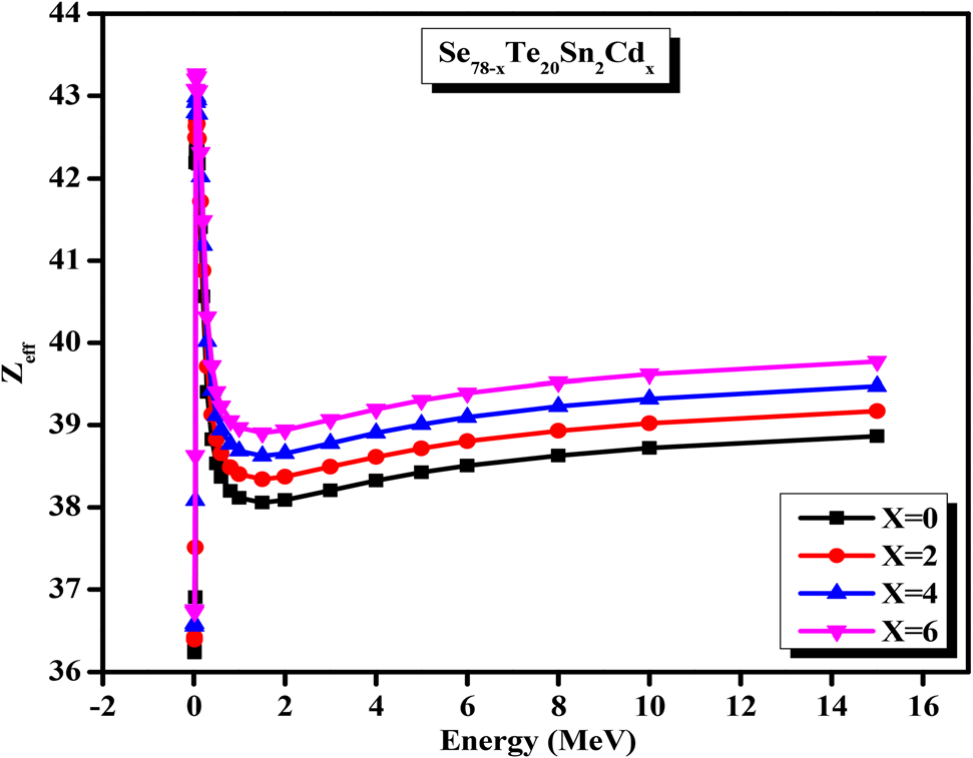
*Z*_eff_ value plots in relation to incoming gamma photon energy for the SeTeSn and SeTeSnCd study.

**Fig. 12:**
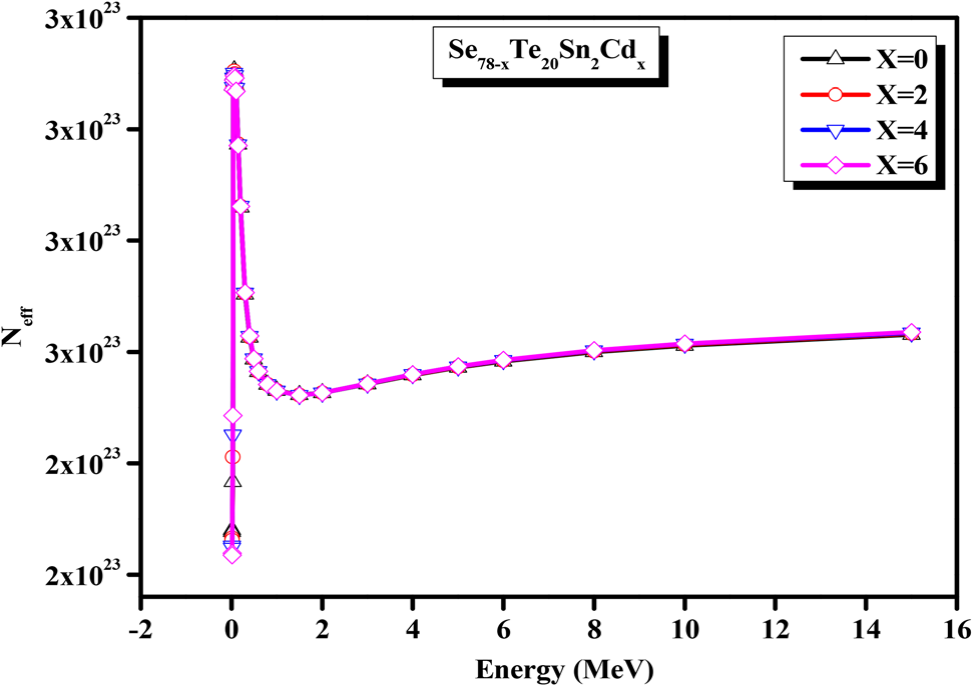
*N*_eff_ value plots in relation to incoming gamma photon energy for the SeTeSn and SeTeSnCd glass system. Theoretical results obtained from deterministic calculations are shown without error bars.

At higher photon energies within the investigated range, HVL and MFP continue to increase gradually. In this region, interaction processes are governed predominantly by Compton scattering, with only a limited contribution from pair production due to the relatively moderate *Z* of the constituent elements. Therefore, the compositional influence of cadmium becomes less pronounced, and the variation in HVL and MFP among samples narrows. The observed trends indicate that cadmium incorporation most effectively enhances attenuation in the photoelectric regime, whereas its effect diminishes as photon energy increases. The atomic cross section (ACS) is the probability that an incoming radiation particle will interact with an atom of the shielding material and is expressed in square meters per atom. Similarly, the electronic cross section (ECS) quantifies the interaction probability per electron and is expressed in square meters per electron. Higher ACS and ECS values indicate stronger photon–matter interactions within the material, thereby enhancing radiation absorption and scattering. As a result, materials exhibiting larger ACS and ECS values are more effective in limiting radiation penetration and provide improved protection against ionizing radiation. Fig. 9 and 10 illustrate the variation of ACS and ECS with photon energy, ranging from 0.015 to 15 MeV, for the samples under study. Both ACS and ECS exhibit higher values at lower photon energies. The ACS and ECS quantify the probability of photon interaction per atom and per electron, respectively. In the present glass system, both parameters exhibit high values in the low-energy region and decrease sharply with increasing photon energy. This trend reflects the dominance of the photoelectric effect at low energies, where interaction probability depends strongly on *Z* and the electronic environment. The incorporation of cadmium increases ACS in the low-energy regime by introducing a higher-atomic-number species into the chalcogenide network. Structurally, cadmium incorporation increases mass density and enhances atomic packing, thereby collectively increasing the probability of interactions per unit volume. The ECS values exhibit comparatively small compositional variation, particularly in the intermediate-energy region, where Compton scattering dominates and interaction probability depends primarily on total electron density rather than *Z*. At higher photon energies, both ACS and ECS values converge for all compositions. This convergence indicates that interaction mechanisms in this region are less sensitive to moderate compositional differences within the studied *Z* range. Thus, cadmium incorporation significantly enhances interaction probability in the photoelectric regime, while its relative influence decreases as higher-energy interaction processes become dominant. Fig. 11 illustrates the relationship between *Z*_eff_ and gamma-ray energy, showing a noticeable decrease in *Z*_eff_ at intermediate and high energies. Fig. 12 illustrates the derivation of the *N*_eff_ from *Z*_eff_, showing that *N*_eff_ variations in response to gamma-photon energy closely mirror those of *Z*_eff_. The *Z*_eff_ represents the weighted atomic response of a multicomponent system to photon interaction and is particularly relevant in the photoelectric energy regime. In the present Se–Te–Sn–Cd glasses, *Z*_eff_ values are highest at low photon energies, reflecting the strong dependence of photoelectric absorption on *Z*. The progressive incorporation of cadmium increases *Z*_eff_ by introducing higher-*Z* species into the amorphous network. This compositional modification enhances the probability of photon interaction in the low-energy region, where *Z* plays a dominant role. As photon energy increases, *Z*_eff_ decreases and the differences between compositions become less pronounced. This behaviour arises from the transition to Compton scattering, which depends primarily on total electron density rather than strongly on *Z*. In this intermediate-to high-energy region, structural densification from cadmium incorporation still contributes to the overall interaction probability; however, the sensitivity to *Z* differences is significantly reduced. The energy-dependent *Z*_eff_ behaviour therefore reflects the combined influence of coordination-driven compositional modification and the governing photon interaction mechanism. The *N*_eff_ follows a similar energy-dependent trend because it is derived from *Z*_eff_ and the average atomic composition. The modest compositional variation in *N*_eff_ across the series indicates that, although cadmium increases mass density and *Z*, the total electron population per unit mass does not change substantially. Consequently, compositional effects are most significant in the photoelectric regime and progressively diminish at higher photon energies.

**Fig. 13:**
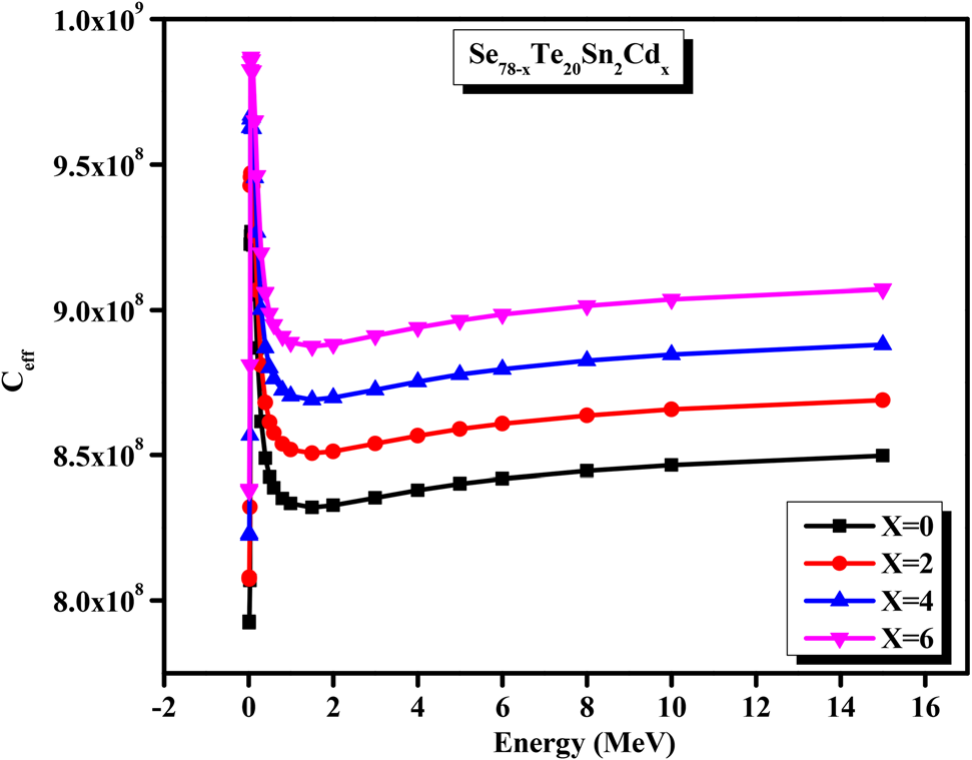
*C*_eff_ value plots in relation to incoming gamma photon energy for the SeTeSn and SeTeSnCd glassy system. Theoretical results obtained from deterministic calculations are shown without error bars.

**Fig. 14:**
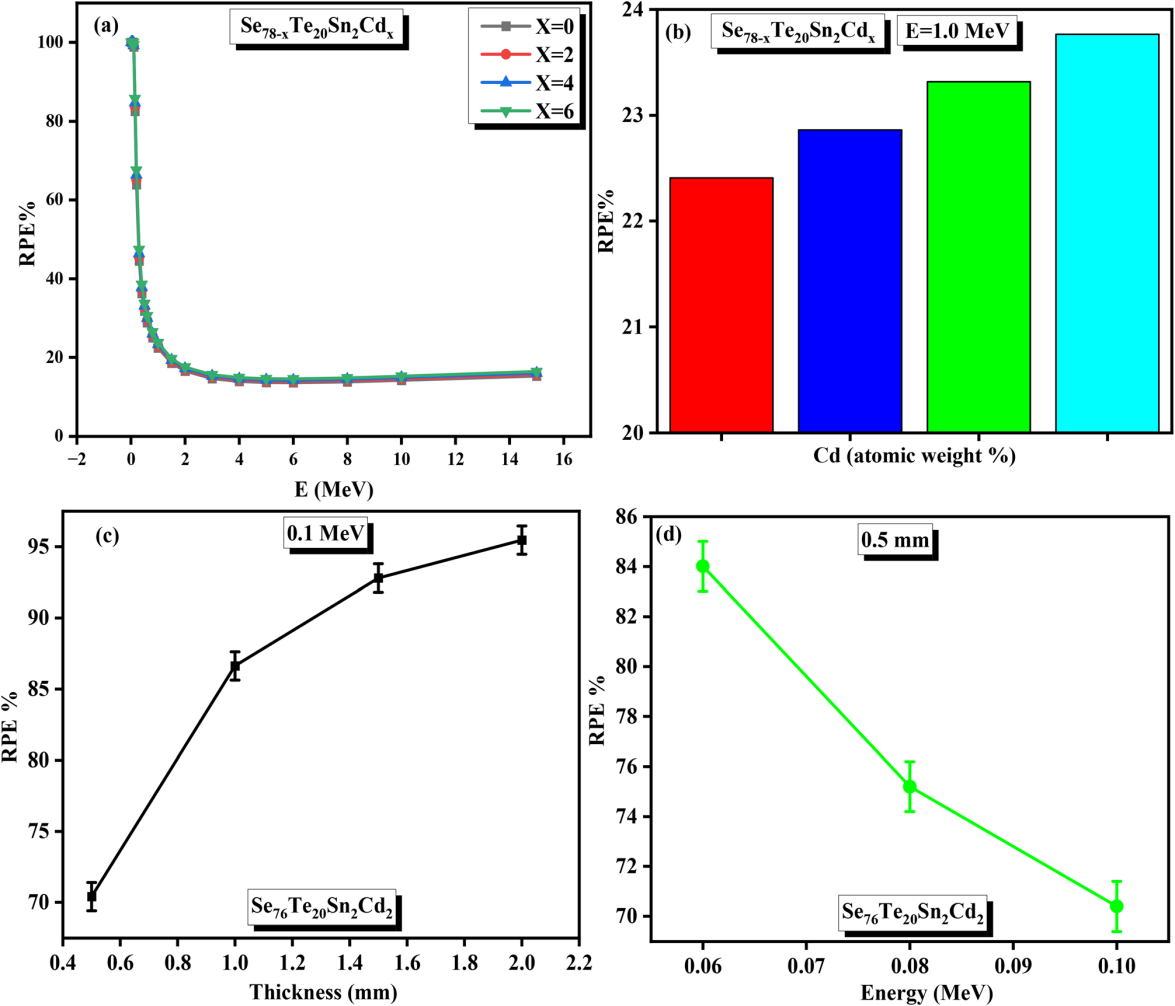
Variation of RPE with (a) incident gamma photon energy and (b) Cd composition (*x*). (c) Thickness-dependent experimental RPE (%) at a constant energy of 0.1 MeV, and (d) energy-dependent experimental RPE (%) at a constant thickness of 0.5 mm. Error bars represent the standard error of the mean calculated from five repeated measurements (*n* = 5). Theoretical results obtained from deterministic calculations are shown without error bars.

**Fig. 15:**
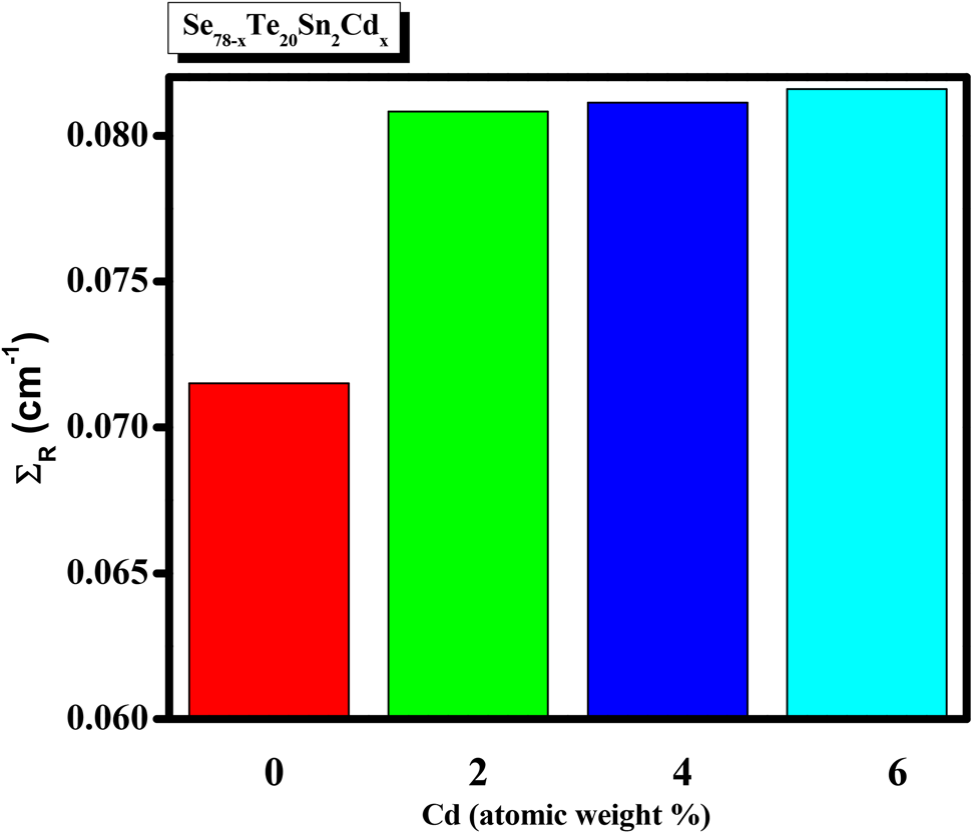
Effective cross-section concentration dependency for rapid neutron removal Σ*R*. Theoretical results obtained from deterministic calculations are shown without error bars.

**Fig. 16:**
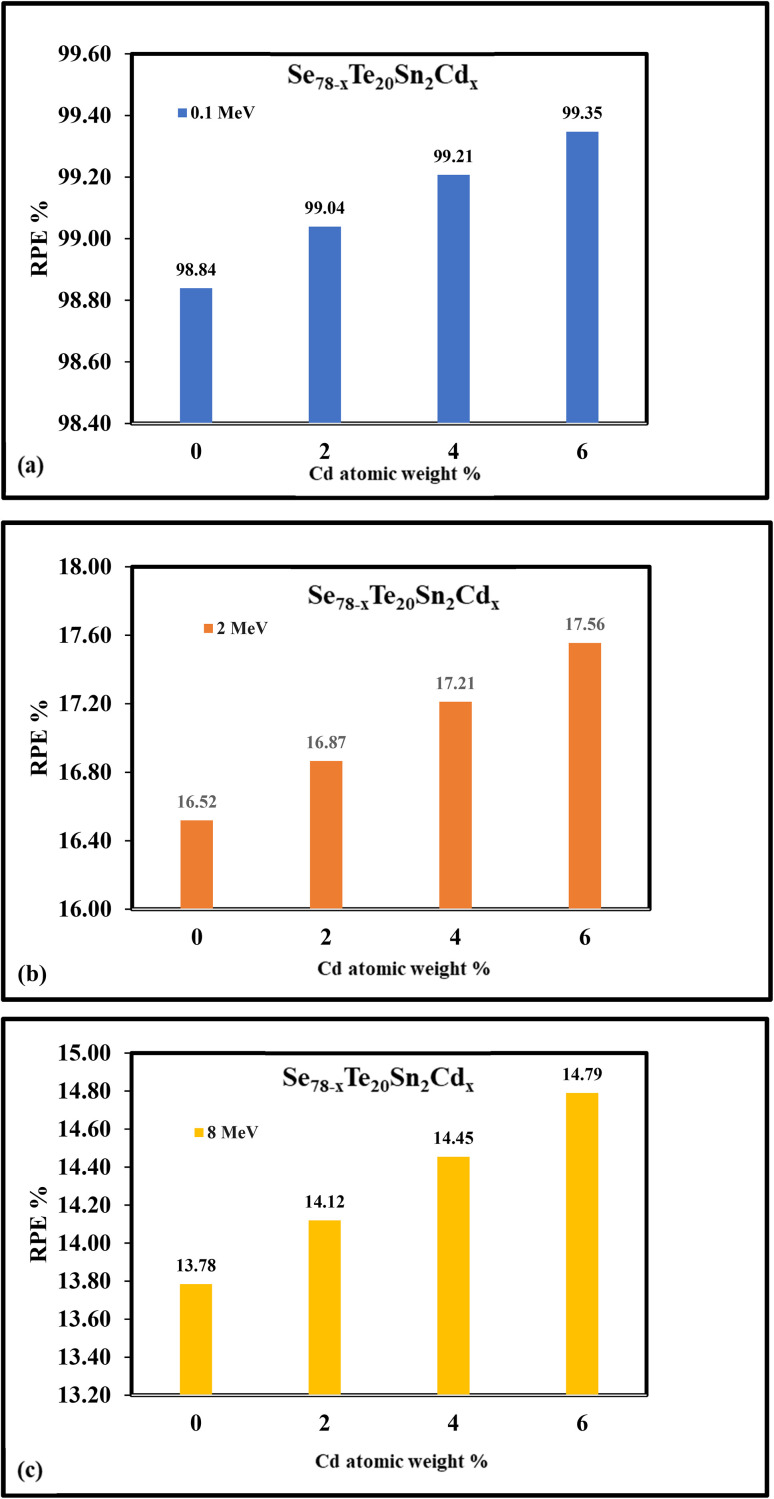
Variation in radiation protection effectiveness (RPE%) according to sample makeup. At (a) low, (b) medium, and (c) high input photon energy, the RPE is assessed. When the sample thickness is *x* = 1.0 cm, the RPE is calculated.

Additionally, for the current STSC ChGs, the effective conductivity (*C*_eff_), an essential metric in radiation protection research, has been calculated and is presented in Fig. 13. The cadmium levels significantly influence *C*_eff_ values, with increased cadmium concentration leading to higher *C*_eff_ values. Specifically, *C*_eff_ values show a substantial increase from STSC (*x* = 0) to STSC (*x* = 6), indicating that composite composition significantly affects the *C*_eff_ trend. For instance, at 0.1 MeV, *C*_eff_ values range from 9.22 × 10^8^ to 9.46 × 10^8^ s m^−1^ as the cadmium concentration increases. As illustrated in Fig. 13, both energy and material composition significantly influence the *C*_eff_ trend, with *C*_eff_ values starting low and rising with increasing gamma photon energy. These findings underscore the importance of considering the *Z*_eff_ and conductivity when designing chalcogenide-based materials for radiation shielding applications.^59–63^ RPE, commonly expressed as RPE = 1 – TF, varies, as shown in Fig. 14(a) and (b), and is a key parameter in assessing the effectiveness of shielding materials against ionizing radiation. In the low-energy photon region, RPE values approach 100%, indicating near-total attenuation of incoming radiation. However, as photon energy increases, RPE decreases, reflecting the material's reduced ability to attenuate at higher energies. Among the STSC glassy alloys studied, the sample with the highest cadmium content (STSC with *x* = 6) exhibits the highest RPE values at 1.0 MeV, as clearly shown in the bar graph in Fig. 14(b), underscoring cadmium's role in enhancing radiation shielding performance. Furthermore, in Fig. 14(c), the relationship between material thickness and RPE is also noteworthy. At a constant photon energy of 0.1 MeV, increasing the thickness of the shielding material increases the RPE, demonstrating that thicker materials provide better protection by absorbing more radiation. Conversely, in Fig. 14(d), at a constant thickness of 0.5 mm, RPE decreases with increasing photon energy, indicating that higher-energy photons are less effectively attenuated by the same material thickness. The RPE of STSC glassy alloys varies with cadmium concentration across photon energies. As depicted in Fig. 14(a) and (b), RPE values increase with higher cadmium content in the material. This trend is consistent across the low-, medium-, and high-photon-energy regions, underscoring the pivotal role of cadmium in enhancing the radiation-shielding capabilities of these alloys. The incorporation of cadmium into the glass matrix enhances the attenuation of incoming radiation, thereby improving the material's protective performance. This enhancement can be attributed to cadmium's high *Z*, which increases the probability of photon interaction and absorption within the material. Consequently, adjusting the cadmium content optimizes the alloy's shielding properties to meet specific application requirements. The bar chart in Fig. 15 illustrates the effective removal cross-section Σ*R* values for each sample, expressed in cm^2^ g^−1^. A consistent increase in Σ*R* is observed with rising cadmium concentration, correlating with the corresponding increases in *V*_m_ and *ρ* (as depicted in Fig. 3). These variations in Σ*R*, along with changes in mechanical and physical properties, provide valuable insights for designing glass compositions that meet gamma-ray shielding requirements and technical specifications for targeted applications.^24,50^Fig. 16 presents the variation in RPE% across different sample compositions and photon energy ranges (low, medium, and high). The results indicate a steady increase in RPE with increasing cadmium content across all photon energy regions, highlighting the significant role of cadmium in enhancing the glass matrix's radiation attenuation capability. Moreover, we compared the results of RPE% at different photon energy with commercial material, which is given in Table 5. Our prepared samples exhibited higher RPE% than existing materials, further confirming their superior shielding effectiveness. This makes them promising candidates for use in diagnostic imaging modalities such as mammography, dental radiography, conventional X-rays, and CT/PET scans, offering enhanced radiation protection for both patients and healthcare professionals.

**Table 5:** Comparison of the theoretical RPE% of the present sample with commercial glasses

Energy (MeV)	Cd_0_	Cd_2_	Cd_4_	Cd_6_	Type A glass	Type B glass	Type C glass	RS 253-G18	RS-360
0.06	100.0	100.0	100.0	100.0	58.0	58.4	40.6	87.7	99.9
0.1	98.8	99.4	99.5	99.6	37.6	37.7	26.8	54.2	100.0
0.5	31.8	35.3	35.6	36.0	19.5	19.5	14.1	20.0	32.6
1	22.4	25.0	25.2	25.5	14.6	14.6	10.5	14.8	16.1
5	13.6	15.4	15.5	15.7	7.1	7.1	4.9	7.2	10.0
10	14.2	16.0	16.2	16.4	6.0	6.0	4.0	6.0	11.4
15	15.2	17.2	17.4	17.6	5.7	5.735	3.8	5.8	12.8

## 5. Conclusions

The present work systematically explored the structural, physical, and radiation shielding characteristics of Se_78−*x*_Te_20_Sn_2_Cd_*x*_ (*x* = 0, 2, 4, and 6) quaternary chalcogenide glasses over a wide photon energy range. XRD and DSC investigations confirmed the predominantly amorphous nature and good thermal stability of all prepared compositions. The incorporation of cadmium significantly modified the local glass structure, resulting in improved atomic packing density and enhanced compactness of the glass matrix. Consequently, the density increased from 4.50 to 4.80 g cm^−3^, while the molar volume decreased from 19.87 to 17.59 cm^3^ mol^−1^ with increasing Cd concentration. The radiation shielding performance was strongly influenced by both photon energy and cadmium content. The gradual increase in MAC and LAC values with increasing Cd concentration demonstrated the beneficial role of cadmium in attenuating low-energy photons. Among all investigated compositions, Se_72_Te_20_Sn_2_Cd_6_ exhibited the best overall shielding performance. In addition, the reduction in TF and the near-complete RPE values observed in the low-energy region confirmed the excellent attenuation capability of the prepared glasses against diagnostic X-ray photons. Although HVL and MFP increased with photon energy due to reduced interaction probability at higher energies, the investigated glasses still maintained promising shielding efficiency within the diagnostic energy range. The enhancement in ACS, ECS, *Z*_eff_, *N*_eff_, and *C*_eff_, further supported the improved photon interaction probability after Cd incorporation. Moreover, the effective electrical conductivity slightly increased with increasing cadmium concentration, indicating additional modifications in the electronic structure of the glass system. A comparative assessment revealed that the prepared Cd-doped chalcogenide glasses demonstrated superior shielding behaviour compared with several conventional commercial shielding materials such as RS-360, RS-253G18, and Types A, B, and C, even at lower thicknesses. These findings highlight the potential of the developed glasses as lightweight, efficient shielding materials for diagnostic radiation protection applications in the 20–150 keV energy range. However, the present study is primarily limited to theoretical and experimental evaluations of photon-shielding behaviour under laboratory-scale conditions. Mechanical durability, long-term environmental stability, thermal ageing behaviour, and large-scale fabrication feasibility were not investigated in the current work. In addition, the shielding performance against neutron and high-energy charged particle radiation remains unexplored. Therefore, future studies should focus on multifunctional optimization of these glass systems by examining their mechanical strength, chemical stability, biocompatibility, and real-device applicability under practical medical and industrial radiation environments. Further compositional engineering and nanostructural modifications may also provide an effective route toward developing next-generation lightweight radiation-shielding materials with enhanced multifunctional performance.

## Author contributions

Vishnu Saraswat: data curation, theoretical and experimental analysis, writing – original draft, S. D. Sharma and R. K. Chaudhary: experiment performed, Z. Khattari: formal analysis, N. Mehta: writing – review & editing, supervision.

## Conflicts of interest

The authors declare no conflicts of Interest.

## Data Availability

The data supporting the findings of this study are available within the article. Additional datasets generated or analysed during the current study are available from the corresponding author on reasonable request.
